# The maxispan procedure makes the phonological similarity effect disappear while increasing recall performance

**DOI:** 10.3758/s13423-024-02594-1

**Published:** 2024-10-10

**Authors:** Simon Gorin, Valérie Camos, Pierre Barrouillet

**Affiliations:** 1 Faculté de Psychologie et des Sciences de L’Éducation, Université de Genève, 40 Boulevard du Pont d’Arve, 1200 Genève 4, Genève, Switzerland; 2 Département de Psychologie, Université de Fribourg, Fribourg, Switzerland

**Keywords:** Working memory, Maxispan procedure, Phonological similarity effect

## Abstract

Based on the hypothesis of two maintenance mechanisms of verbal information in working memory, an articulatory loop and an attentional executive loop, Barrouillet et al. predicted and observed that facilitating the optimal use and separation of these two systems results in a strong increase in recall performance. They developed for this purpose the maxispan procedure, in which participants cumulatively rehearse aloud a limited number of the first items of the series (i.e., three or four) and keep rehearsing them until the end of the series before recall. Beyond increasing recall performance, the model also predicts that the maxispan procedure should also abolish the phonological similarity effect (PSE, the poorer recall of phonologically similar than dissimilar items) in both the rehearsed and the nonrehearsed items by permitting the perfect maintenance of the former in a nonoverloaded articulatory loop and preventing storage of phonological traces of the latter in the attentional system. However, the PSE should reappear if too many items are verbally rehearsed in the maxispan procedure. In this case, the overload of the articulatory loop should lead to offload its content into the attentional system where phonologically similar traces are prone to confusion. We tested and verified these hypotheses in two experiments.

The ability to immediately reproduce a verbal input was among the first objects of study in psychology (Ebbinghaus, 1885; Holmes, 1871), and the measurement of this capacity was part of the very first psychological tests (Binet & Simon, 1904; Cattel, 1890), probably for its role in language learning, use, and understanding. However, not before the middle of the twentieth century was attention turned by Broadbent’s (1958) and Peterson and Peterson’s (1959) research to what we call short-term memory (STM), eliciting a series of theoretical proposals that Atkinson and Shiffrin (1968) suggested to synthesize in a “modal model.” It was by questioning the capacity of the short-term store of this model to fulfill the functions of a working memory (WM) coordinating processing and storage that Baddeley and Hitch (1974) laid the foundations of their multicomponent model in which verbal STM is conceived as a phonological loop.

To account for their findings, Baddeley and Hitch (1974) initially supposed the existence of a phonemic loop in which a small amount of verbal material (e.g., three or four digits) could be maintained through articulatory rehearsal. When the capacity of this loop was exhausted, a central workspace that could meet storage as well as processing demands was assumed to store the supernumerary items. Further theoretical elaborations restricted verbal storage function to the peripheral system called a phonological loop, in which a phonological store would hold speech-based memory traces that suffer from temporal decay and are lost after about 1.5 s or 2 s (Baddeley, 1986). However, these traces could be reactivated by an articulatory loop that could read them off—and feed them back into—this store. Verbal memory spans would thus correspond to the amount of verbal material individuals are able to articulate in 2 s. Within this theoretical framework, the central workspace called the central executive would be deprived of any storage capacity. This strict distinction has been recently attenuated in Baddeley’s multicomponent model by assuming the existence of an episodic buffer that could store multimodal and potentially verbal information maintained through attentional refreshing (Baddeley et al., 2021).

The hypothesis of two maintenance mechanisms of verbal information in WM is one of the tenets of the time-based resource-sharing model (Barrouillet & Camos, 2015, 2021). Camos et al. (2009) gathered evidence that verbal information can be maintained through articulatory rehearsal in a phonological loop, as in Baddeley’s model, or through attentional refreshing in an executive loop in charge of both processing and storage (see Camos, 2017, for review). Further evidence of this dual structure was recently provided by a new procedure in immediate serial recall (ISR) we call the maxispan procedure (Barrouillet et al., 2021). This procedure was inspired by the fact that adding the estimated capacities of the two maintenance systems (i.e., the executive and the phonological loop, four letters in both cases; Cowan, 2001; Vergauwe et al., 2014) largely exceeds adult’s verbal STM for letters, which is about six (Dempster, 1981). We reasoned that this was because individuals, naturally unaware of its dual structure, misuse their verbal WM by rehearsing too many items. Overloading the articulatory loop would cause the temporary offloading of some items into the executive loop from which they can be retrieved for rehearsal. This back and forth between the two systems might lead to the loss of part of the information. In order to facilitate the optimal functioning of the two systems by their separation, we designed an ISR task in which participants were presented with series of letters. They were asked to cumulatively rehearse aloud the three or four first letters presented, and to keep rehearsing them while trying to memorize the following letters. Limiting the number of letters rehearsed should avoid overloading the phonological loop, and their rehearsal until the end of the series would prevent the following letters to enter this loop. Moreover, this articulatory maintenance, involving minimal attentional demand, would preserve attentional resources, favoring a more efficient storage of the following letters in the executive loop. As we predicted, the maxispan procedure resulted in a dramatic increase of letter spans compared with the traditional simple span procedure, the rehearsed, but also the nonrehearsed letters, being better recalled than in a traditional simple span procedure.

However, beyond the increase of recall performance, the hypothesis of a dual system of verbal maintenance predicts other effects related to the maxispan procedure, such as the disappearance of the phonological similarity effect (PSE; Baddeley, 1966; Conrad, 1964). In ISR tasks, lists of phonologically similar items (e.g., *cat, map, man, cap, mad*) lead to poorer recall than phonologically dissimilar items (e.g., *pit*, *day*, *cow*, *pen*). This effect is classically attributed to a coding or recoding of verbal information in a phonological format, and to its maintenance in some phonological buffer (Baddeley, 1986). Accordingly, the PSE disappears under articulatory suppression, when participants are required to repeatedly say something like the word “*the*” (Estes, 1973; Murray, 1968). Occupying the articulatory system prevents the encoding of memoranda in a phonological format when presented visually, as well as their maintenance by subvocal rehearsal.

Why should the PSE disappear under the maxispan procedure? Because the number of letters rehearsed (up to four) does not exceed the capacity of the articulatory loop, and, because these letters are rehearsed aloud until the end of the series, they are virtually perfectly recalled in the maxispan procedure (Barrouillet et al., 2021). Because the phonological similarity of these letters does not increase articulatory demands, their rehearsal should not exceed the articulatory capacities of adults, and they should be perfectly recalled. Thus, the PSE should not affect the rehearsed letters. Moreover, articulating these first letters until the end of the series prevents the phonological encoding of the following letters, those that are not rehearsed. We hypothesize that these nonrehearsed letters are consequently stored in the executive loop in some nonphonological format (e.g., visual, or semantic through idiosyncratic associations), and maintained through attentional refreshing. Thus, the following nonrehearsed letters as well as the rehearsed letters should remain immune to PSE, making this effect disappear. Interestingly, and contrary to other manipulations like articulatory suppression that make the PSE disappear, note that the maxispan procedure should lead to a better, and not a lower, overall recall performance. These hypotheses were tested in Experiment 1.

## Experiment 1

### Method

#### Experimental design

The experiment relied on a 2 (procedure: simple span vs. maxispan) × 2 (phonological similarity: similar vs. dissimilar) × 7 (serial position: 1 to 7) mixed design, with procedure as a between-participant variable and phonological similarity and serial position as a within-participant variables. In the maxispan group, we instructed participants to cumulatively rehearse the first four letters, presented in blue, until recall and to mentally maintain the following black letters. No particular instructions on how to maintain the letters during the trials were provided to the participants in the simple span procedure group.

#### Participants

Forty students from the University of Geneva took part in the experiment in exchange of partial course credits. They were tested individually, and alternatively assigned to each of the two procedures, starting with the maxispan group, each group including 20 participants. The experiment has been approved by the local ethics committee of the Faculty of Psychology and Sciences of Education of the University of Geneva, and all participants gave their informed consent to participate.

#### Sampling plan

We used a Bayesian open-ended sequential design (Schönbrodt & Wagenmakers, 2018) as a sampling plan procedure. We conditioned the decision to stop data collection to the results of the main analysis (i.e., the comparison of recall accuracy between the maxi and simple span groups). Based on evidence from past studies that the maxispan procedure is characterized by a very large effect, we planned to first collect a minimum of 20 participants in each group before performing the main analysis (see the Results section for more details). If for the main effect of procedure and the interaction between procedure and similarity the results provided substantial evidence supporting their presence (BF_10_ > 3) or their absence (BF_01_ > 3), data collection was stopped. If not, the plan was to collect more participants by batches of five in each group until the desired level of evidence was obtained to take informed decision with regard to the presence or the absence of the predicted effects.

#### Material

The material consisted of 44 lists composed of seven consonants. One half was composed of phonologically similar consonants and the other half of phonologically dissimilar consonants. The lists with similar letters were generated using the consonants “B,” “C,” “D,” “G,” “P,” “T,” and “V,” which all share the same ending sound in French. For the lists with dissimilar letters, we used the consonants “F,” “J,” “K,” “Q,” “R,” and “S” and one of the three consonants “L,” “M,” and “N” randomly selected. This was done to avoid that these last three consonants, which are to some extent phonologically similar to each other, induced phonological similarity inside lists of dissimilar items. For each participant, we randomly chose which of the letters “L,” “M,” and “N” completed the pool of dissimilar letters, and the same seven dissimilar consonants were used in all the trials.

For each condition of phonological similarity, the lists were generated by shuffling the corresponding seven letters, and the order of the lists was defined at random. We ensured that no successive consonants in the lists were alphabetically adjacent (e.g., “B” followed by “C” was not allowed), and controlled that the same consonant was not presented at the same serial position in two consecutive trials. Finally, we generated 20 sets of 44 lists, with the constraints described above. To ensure a strict comparability between the two procedures, the same sets of 44 lists were used in both maxi and simple span procedure groups.

#### Procedure

**Fig. 1:**
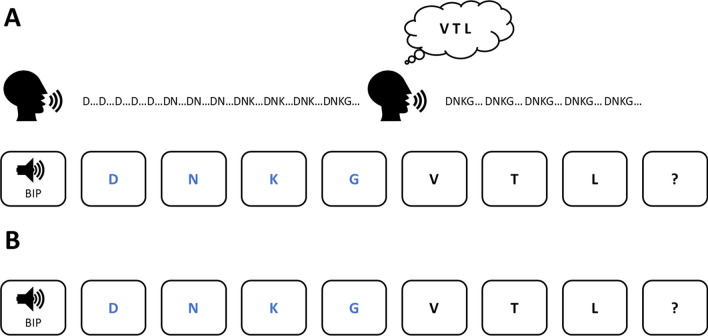
Illustration of an experimental trial in the maxispan (**A**) and the simple span (**B**) procedures in Experiment 1

The experimental task was built using the open-source software OpenSesame (Mathôt et al., 2012), and the same procedure as described in Barrouillet et al. (2021) was followed (Fig. 1). Each trial was initiated by the participant and started with a 440-Hz tone played for 500 ms to indicate the start of the trial. After a silent period of 500 ms, the letters were displayed sequentially at the center of the screen, following a presentation rate of one item every 2 s (1.5 s on, 0.5 s off). It is important to emphasize that this slow presentation rate was used to allow participants to rehearse up to four to-be-rehearsed letters between each item presentation. The first four letters to be cumulatively rehearsed in the *maxispan* group were displayed in blue, while the three other letters were displayed in black font.

Directly after the presentation of the last letter, recall was prompted by showing a question mark at the center of the screen. Participants were instructed to recall the letters orally in their order of presentation, and they could skip a serial position by saying “blank” if they forgot the corresponding item and the experimenter wrote down the response for latter transcription. The next trial was initiated by the participant by pressing the space bar of the computer keyboard.

Participants in the simple span procedure group were instructed to remember the letters for further serial recall without paying attention to their color. The participants in the maxispan procedure group were asked to cumulatively rehearse the blue letters aloud during the presentation of the letters, and to continue the aloud rehearsal of these blue letters during the presentation of the black letters while maintaining these black letters in memory but without using rehearsal. These instructions were presented to the participants along with a figure (see Fig. 1A and B for maxispan and simple span procedure groups, respectively) used by the experimenter to explain and demonstrate the procedure. After the demonstration by the experimenter, the participants performed four practice trials to familiarize them with the experimental procedure and to ensure that they understood instructions. The practice trials were of critical interest to verify the perfect understanding of the task requirements and the cumulative rehearsal method. After the practice, the participants performed the 40 self-paced experimental trials.

#### Scoring procedure

Participants’ responses were scored applying the following procedure. For each list, items recalled at the correct serial position were scored as correct (1) and items not recalled or recalled at the wrong serial position, were scored as incorrect (0).

### Results

The statistical analyses were conducted using the open-source statistical software JASP 0.18.3 (JASP Team, 2024), with the default priors. The main analysis focused on the effect of procedure, phonological similarity and serial position on recall accuracy. To this aim, we conducted a Bayesian mixed analysis of variance (ANOVA) on the proportion of letters correctly recalled, with procedure as a two-level between-subject variable (maxi vs. simple span), phonological similarity as a two-level within-subject variable (similar vs. dissimilar), and serial position as a seven-level within-subject variable (from 1 to 7). Given the larger number of the models involved in the analysis, we directly present the results of the analysis of effects (matched models).

As expected, the analysis provided decisive evidence for an effect of procedure, BF_10_ = 2.06 × 10^6^, recall performance being higher in the maxispan than in the simple span group (rate of correct recall of 0.88 and 0.64, respectively). There was also substantial evidence for the interaction of this effect with similarity, BF_10_ = 3.09, as well as decisive evidence for the effect of serial position and its interaction with all the other effects including the Similarity × Condition interaction (all BF_10_ > 100). Given the strong support for the three-way interaction, we analyzed the effect of phonological similarity and serial position separately for each group.

**Fig. 2:**
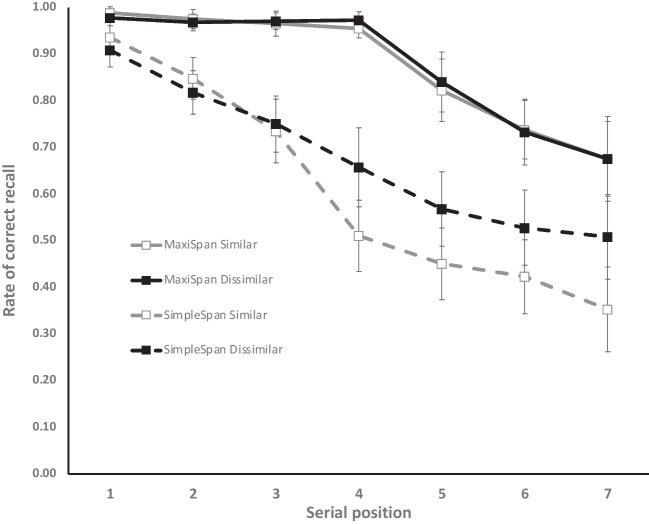
Rate of correct recall as a function of the procedure (maxispan vs. simple span) and serial position in Experiment 1. Error bars represent 95% confidence intervals

Concerning the simple span group, dissimilar lists were better recalled than similar lists (0.68 and 0.61, respectively), BF_10_ = 4.71, an effect that interacted with serial position, BF_10_ = 7.70 × 10^6^. The similarity effect was absent from the three first serial positions, but large in the four latest (Fig. 2). By contrast, as we predicted, the similarity effect disappeared in the maxispan group with substantial evidence for the null, BF_01_ = 4.82, as well as for the absence of interaction between similarity and serial position, BF_01_ = 39.32. Recall in the maxispan group followed the expected serial position curve with a virtually perfect recall for the rehearsed blue letters, whereas recall rate of the following letters progressively decreased as serial position increased (Fig. 2), a pattern that contrasted with the serial position curve in the simple span procedure, leading to the Procedure × Serial Position interaction already mentioned.

### Discussion

As we predicted, the maxispan procedure not only resulted in better recall performance but also abolished the PSE for the rehearsed as well as for the nonrehearsed letters. In a tentative account of the maxispan and the simple span procedures, following an old hypothesis put forward by Baddeley et al. (1975) and subsequently abandoned, Barrouillet et al. (2021) assumed that the information maintained in the so-called phonological loop is articulatory and not phonological in nature. The loop would maintain articulatory programs activated either by reading the letters when visually presented or listening to them,^1^ while this reading or listening results in phonological traces in the executive loop due to the attention paid to the letters to memorize. Barrouillet et al. (2021) hypothesized that when several letters follow each other, as in the maxispan procedure, these motor programs are concatenated with each other to form an articulatory object (e.g., “BTD” articulated as “beeteedee”) that consolidates through repetition. Rapidly, this articulatory object no longer needs attention for its articulation, and the phonological traces stored in the executive loop decay, leaving attention free for storing the following letters. However, this is only possible if the complexity and length of the motor program does not exceed the capacity of the articulatory loop. Barrouillet et al. (2021) observed that this limit was four for letters, adding a fifth to-be-rehearsed letter modifying the recall pattern of maxispan.

In the simple span procedure, because the presentation of the letters triggers the automatic activation of the corresponding motor programs via a kind of affordance (Jones & Macken, 2018), individuals tend to rely naturally on rehearsal for maintaining the memoranda. However, when the number of letters to be memorized exceeds the capacity of the articulatory loop, the motor program breaks down, resulting in a chaotic rehearsal schedule in which phonological traces of the letters are reintroduced into the executive loop by their articulation—participants hearing their own pronunciation, to which they necessarily pay attention. Verbal rehearsal, instead of running a motor program in an articulatory loop with a minimal attentional demand, relies now on successive searches and retrievals of these phonological traces from the attentional system, the capacity of which is rapidly reached, hence the loss of some memory items and poorer recall.

Such a model predicts that a maxispan procedure in which participants would be asked to rehearse a number of letters exceeding the articulatory loop capacity (e.g., six letters) should involve the same back-and-forth flow of information as in the simple span procedure, abolishing the benefit of the maxispan procedure, including the reappearance of the PSE. Indeed, articulating the letters introduces phonological representations into the executive loop, from which these representations are searched and retrieved for rehearsal attempts. This should lead to confusions when letters are phonologically similar. Consequently, the PSE should reappear in the maxispan procedure as it is observed in simple span procedures. This hypothesis was tested in Experiment 2.

## Experiment 2

### Method

#### Participants

Forty students from the University of Geneva were recruited to take part in the experiment in exchange for partial course credits. They were tested individually and alternatively assigned to each of the two procedures, starting with the maxispan group; each group included 20 participants. The experiment has been approved by the local ethics committee of the Faculty of Psychology and Sciences of Education of the University of Geneva, and all participants gave their informed consent to participate. None of them took part in the first experiment. One of the participants in the maxispan group, a nonnative French speaker, was discarded from the study. Analyses in this group were thus performed with 19 participants.

#### Procedure

Design, material, sampling plan, scoring, and procedure were the same as in Experiment 1, with the only exception being that in each list of seven letters, the first six letters, instead of four, were in blue font, and participants in the maxispan group were asked to cumulatively rehearse these six blue letters. The last letter was in black font.

### Results and discussion

**Fig. 3:**
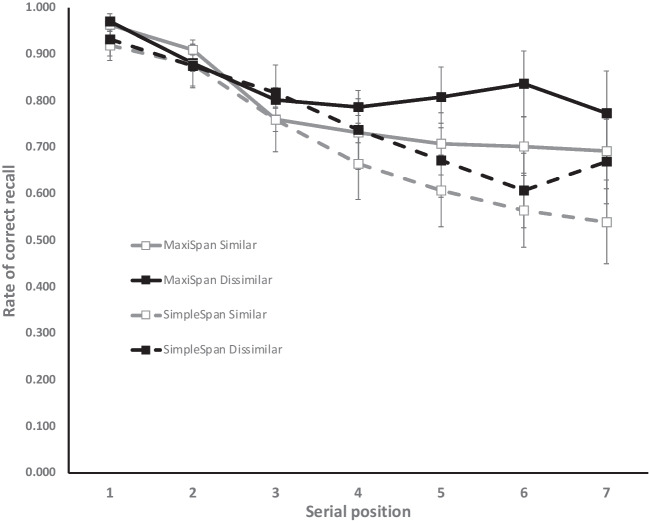
Rate of correct recall as a function of the procedure (maxispan vs. simple span) and serial position in Experiment 2. Error bars represent 95% confidence intervals

The same analyses as in Experiment 1 were performed on the rate of letters correctly recalled. Apart from an effect of serial position, BF_10_ = 5.25 × 10^30^, and more importantly for the aim of the study, the analyses revealed very strong evidence for an effect of similarity, BF_10_ = 56.64, recall performance being higher with dissimilar than similar lists (0.80 and 0.74, respectively). This effect interacted with serial position, BF_10_ = 84.27 × 10^2^, the effect being stronger in the last than the first position (Fig. 3). However, and in line with our expectation, there was substantial evidence for an *absence* of interaction between similarity and procedure, BF_01_ = 3.15, and anecdotal evidence for the absence of the triple Similarity × Procedure × Serial Position interaction, BF_01_ = 1.71. Whereas there was decisive evidence for an effect of procedure in Experiment 1, the evidence here was only anecdotal, BF_01_ = 1.43, though recall performance was still descriptively higher in the maxispan than the simple span procedure (0.81 and 0.73, respectively). This difference was more pronounced in the four last serial positions, as testified by the Procedure × Serial Position interaction, BF_10_ = 93.16.

Thus, as we predicted, extending the list of letters to be rehearsed from four to six made the similarity effect reappear in the maxispan procedure as it exists in the simple span procedure. The overall effect of the maxispan procedure on recall performance was not abolished, but strongly reduced and no longer reliable. Thus, this second experiment shed light on the boundaries of the maxispan effect as well as on the determinants of the PSE. These points are discussed below.

## General discussion

The present findings have implications for both the maxispan and the PSEs and their underpinnings, as well as for the structure of verbal STM. First of all, Experiment 1 replicated the maxispan effect previously observed by Barrouillet et al. (2021). Rehearsing a number of items within the capacity limits of the articulatory loop facilitates recall not only of the rehearsed but also of the nonrehearsed items (see Serial Positions 5, 6, and 7 in Fig. 2). Interestingly, as we predicted, this benefit at recall is strongly reduced by increasing the number of items to be rehearsed. This confirms Barrouillet et al.’s (2021) intuition that failures to find a beneficial effect of verbal rehearsal, as in Souza and Oberauer (2018), are due to an overload of the articulatory loop by requiring participants to rehearse too many items (six words, with several of them being multisyllabic in Souza & Oberauer’s study). Contrary to what Lewandowsky and Oberauer (2015) have claimed, verbal rehearsal does not seem to be an epiphenomenon but rather an efficient mechanism for maintaining verbal information, provided that its use does not exceed its capacities.

We hypothesized that limiting the number of rehearsed letters should lead to their quasiperfect recall, something that happened, and preserve the capacity of attention to maintain memory traces of the remaining (black) letters within the executive loop. It is worth mentioning that the mean recall rate of these three black letters in the maxispan procedure, despite the fact that they were stored under concurrent articulation, was higher than the mean recall rate of the entire lists (0.75 vs 0.64), and far higher than the corresponding letters (0.47) in the simple span procedure. In other words, these black letters were far less affected by the continuous articulation of the blue letters during their encoding and maintenance in the maxispan procedure than by the previous encoding and maintenance of these blue letters in the simple span procedure. This suggests that, as we assumed, blue and black letters are maintained in distinct stores in the maxispan procedure.

This procedure also made the PSE disappear. Interestingly, this disappearance was accompanied by an increased recall performance, contrary to what happens under articulatory suppression that abolishes the PSE when items are visually presented, but at the cost of poorer recall performance (Estes, 1973; Richardson et al., 1980). The rehearsed items being perfectly recalled are not affected by PSE, and the absence of PSE for the black letters suggests that they were not stored in a phonological format.^2^ Moreover, keeping the respective contents of the articulatory loop and the executive loop separate prevents the occurrence of interference likely to cause the PSE. However, as we predicted, increasing the number of rehearsed letters made the PSE reappear. Of the two systems we assume are at play in maintaining information (the articulatory and the executive loop), which is responsible for the PSE?

A first possibility is that it comes from the executive loop. Rehearsing six instead of four items leads to a breakdown of the articulatory program testified by a poorer recall of these items (from 0.97 to 0.82). Rehearsal in this case would require retrieving from the executive loop memory traces of the successive items for their articulation, this articulation reinforcing in turn the phonological features of their representations. This makes the maxispan akin to what probably happens in a simple span procedure, hence the reappearance of the PSE. Nonetheless, this executive loop must be distinguished from the concept of phonological store hypothesized by Baddeley (1986) and still present in the most recent formulations of the model (e.g., Baddeley et al., 2021). There is ample evidence that the maintenance of verbal information involves attentional resources because it disrupts, and is disrupted by, concurrent attention-demanding tasks, either visual or verbal in nature (Barrouillet et al., 2011; Vergauwe et al., 2010, 2014), as well as concurrent maintenance of visual information (Uittenhove et al., 2019). All these findings are at odds with the hypothesis that verbal information is maintained in a domain-specific phonological store fueled by its own resources, such that the phonological loop is supposed to be. A general system storing multimodal representations, like the episodic buffer (Baddeley, 2000) or the executive loop (Barrouillet & Camos, 2015, 2021), is sufficient in accounting for the PSE if memory traces are phonological representations, something that occurs automatically for verbal material presented auditorily, but also visually (Rubenstein et al., 1971).

Nonetheless, it might be that the articulatory loop, even if it maintains motor programs instead of phonological representations, contributes also to the effect. It is worth noting that before having been attributed to confusion between representations in a phonological store (Baddeley, 1986), the PSE was attributed to confusions between motor articulatory programs that are necessarily akin for phonologically similar items. This hypothesis came from the analyses of recall errors (Hintzman, 1967) and the observation that children deaf from birth, but good speakers, presented a PSE in the recall of lists of consonants (Conrad, 1970). It might be that reactivating memory traces through articulation leads to confusions when several similar motor programs have been recently activated.

To conclude, the dramatic increase in recall performance produced by the maxispan procedure, the resulting abolition of the PSE, and its reappearance when the number of to-be-rehearsed items is increased can all be explained by assuming that verbal STM relies on two independent systems of maintenance—an articulatory loop, running motor programs, along with an executive loop, storing and maintaining multimodal representations through attention.

## Data Availability

The data sets, scripts for data processing and figures generation, as well as statistical output files generated during the current study are available via the Open Science Framework (https://osf.io/s2ptv/).

## Footnotes

1 Neurological studies have shown that the perception of speech activates the motor areas involved in speech production (e.g., Wilson et al., 2004).

2 Due to the ceiling effect for blue letters in the maxispan procedure, it is difficult to draw firm conclusions from the absence of PSE about the format in which they are stored.
